# Design considerations for massively parallel sequencing studies of common familial cancers

**DOI:** 10.1186/1897-4287-10-S2-A38

**Published:** 2012-04-12

**Authors:** BJ Feng, SV Tavtigian, MC Southey, DE Goldgar

**Affiliations:** 1 Department of Dermatology, University of Utah School of Medicine, Salt Lake City, UT, USA; 2 Huntsman Cancer Institute and Department of Oncological Sciences, University of Utah, Salt Lake City, UT, USA; 3 Department of Pathology, University of Melbourne, VIC 3010, Australia

## 

Massively Parallel Sequencing (MPS) allows sequencing of entire exomes and genomes to now be done at reasonable cost, and its utility for identifying genes responsible for rare Mendelian disorders has been demonstrated. However, for a complex disease such as the common cancers, study designs need to accommodate substantial degrees of locus, allelic, and phenotypic heterogeneity, as well as complex relationships between genotype and phenotype. Such considerations include careful selection of samples for sequencing and a well-developed strategy for identifying the few “true” disease susceptibility genes from among the many irrelevant genes that will be found to harbor rare variants. To examine these issues we have performed simulation-based analyses in order to compare several strategies for MPS sequencing in complex disease. Factors examined include genetic architecture, sample size, number and relationship of individuals selected for sequencing, and a variety of filters based on variant type, multiple observations of genes and concordance of genetic variants within pedigrees. A two-stage design was assumed where genes from the MPS analysis of high-risk families are evaluated in a secondary screening phase of a larger set of probands with more modest family histories. Designs were evaluated using a cost function that assumed the cost of sequencing the whole exome was 400 times that of sequencing a single candidate gene. Results indicate that while requiring variants to be identified in multiple pedigrees and/or in multiple individuals in the same pedigree are effective strategies for reducing false positives, there is a danger of over-filtering so that most true susceptibility genes are missed. In most cases, sequencing more than two individuals per pedigree results in reduced power without any benefit in terms of reduced overall cost. Further, our results suggest that although no single strategy is optimal, simulations can provide important guidelines for study design. Examples in familial breast cancer and melanoma will be presented to illustrate these points.

